# Impact of shift work on the diurnal cortisol rhythm: a one-year longitudinal study in junior physicians

**DOI:** 10.1186/s12995-018-0204-y

**Published:** 2018-08-14

**Authors:** Jian Li, Martin Bidlingmaier, Raluca Petru, Francisco Pedrosa Gil, Adrian Loerbroks, Peter Angerer

**Affiliations:** 1 Institute of Occupational, Social and Environmental Medicine, Centre for Health and Society, Faculty of Medicine, University of Düsseldorf, Universitätsstraße 1, 40225 Düsseldorf, Germany; 2 Endocrine Research Unit, Medizinische Klinik und Poliklinik IV, Ludwig-Maximilians-University, Munich, Germany; 3 Institute and Outpatient Clinic for Occupational, Social and Environmental Medicine, WHO Collaborating Centre for Occupational Health, Ludwig-Maximilians-University, Munich, Germany; 4 Clinic for Psychiatry, Psychotherapy and Psychosomatics, Helios Vogtland Clinical Center, Plauen, Germany

**Keywords:** Shift work, Cortisol, Hypothalamic-pituitary-adrenal axis, Longitudinal study, Occupational health

## Abstract

**Background:**

Cumulative epidemiological evidence suggests that shift work exerts harmful effects on human health. However, the physiological mechanisms are not well understood. This study aimed to examine the impact of shift work on the dysregulation of the hypothalamic-pituitary-adrenal axis, i.e. diurnal cortisol rhythm.

**Methods:**

Seventy physicians with a mean age 30 years participated in this one-year longitudinal study. Working schedules, either shift work or regular schedules with day shift, were assessed at baseline. Salivary cortisol samples were collected on two consecutive regular working days, four times a day (including waking, + 4 h, + 8 h, and + 16 h), at both baseline and the one-year follow-up. The diurnal cortisol decline (slope) and total cortisol concentration (area under the curve, AUC) were calculated.

**Results:**

After adjusting for cortisol secretion at baseline and numerous covariates, shift work at baseline significantly predicted a steeper slope (*p* < 0.01) and a larger AUC (*p* < 0.05) of diurnal cortisol rhythm at follow-up in this sample of physicians. In particular, waking cortisol at follow-up was significantly higher among those engaged in shift work than day shift (*p* < 0.01).

**Conclusions:**

Our findings support the notion that shift work changes the diurnal cortisol pattern, and is predictive of increased cortisol secretion consequently in junior physicians.

## Background

Shift work is common in contemporary working life. In the United States, about 29% of the employees had their work time arrangements as shift work, according the 2010 National Health Interview Survey [1]; while the 2010 European Working Conditions Survey indicated that more than 20% workers in Europe were engaged in shift work [2]. In the past decades, several chronic health conditions have been identified to be related to shift work [3]. For example, in 2007 the World Health Organization International Agency for Research on Cancer announced the probable association between shift work and cancer risk [4], particularly breast cancer in women [5] and prostate cancer in men [6]; in addition, it has been observed that shift work increases risk of diabetes [7], myocardial infarction, all coronary events, and ischaemic stroke [8].

Some physiological mechanisms have been proposed to explain links between shift work and adverse health outcomes. Among others, shift work-caused disruption of the circadian time organization is one core explanation, which exerts far-reaching effects at the molecular and cellular levels. Exposure to shift work during one’s occupational career, through the close physiological interaction of circadian clock-related and cell-cycle factors, may result in a variety of processes that initiate epigenetic modifications, with malignant potential. Supportively, expression and methylation of circadian genes, such as BMAL1 and PER1, are evident among shift workers in recent years [9–11]. Meanwhile, melatonin which is produced in the pineal gland and circulated during darkness has received significant attention in shift work research. It exerts broad effects, via specific receptors or entry into cells directly as a small lipophilic molecule, playing a crucial role in regulating the circadian time organization. Commonly co-existing with circadian disruption, “melatonin hypothesis” was formulated in 1987 to link melatonin suppression and cancer risk [12]. To date, much evidence has been gained in the past three decades, on melatonin suppression due to shift work [13, 14]. In addition, shift work is closely related to sleep deprivation and disturbance, causing immune dysfunction as elevated concentrations of C-reactive protein and interleukin 6, and an increase in cellular stress in terms of an altered balance of pro-oxidative and anti-oxidative markers [15].

Regarding the cardiometabolic risk associated with shift work, circadian disruption represents a crucial pathway as well [16, 17]. It has been postulated that circadian disruption induced by shift work would impair the functioning of the hypothalamic-pituitary-adrenal (HPA) axis which regulates the biological response to stressful stimuli [18], while abnormal HPA axis activity increases the risk of subsequent cardiometabolic conditions [19–21]. Cortisol is the most widely studied biomarker of the HPA axis, and it is usually assayed from saliva, serum, urine, or hair [22]. Cortisol shows a strong diurnal rhythm. In the morning cortisol peaks during awakening, then it declines gradually over the day till bedtime [23, 24]. In general, different features of the cortisol diurnal pattern have been frequently examined as indicators of HPA axis function, including the waking cortisol response which is superimposed on the circadian cycle of cortisol release [25], the slope of cortisol decline over the day and the total cortisol concentration over the day such as area under the curve (AUC) [26].

So far, epidemiological studies on shift work and cortisol have found inconsistent, even contrasting, results. For instance, two French studies found that serum cortisol in the evening was increased among workers in night shift [27, 28]. Using urinary or hair cortisol, two studies from the US and the Netherlands respectively observed that shift workers had significantly higher cortisol levels [29, 30]. Also, a British study showed that shift work was associated with higher waking cortisol as well as total AUC in saliva [31]. By contrast, Hung et al. reported lower cortisol AUC in shift workers [32]. In addition, five studies indicated negative associations between shift work and waking salivary cortisol [13, 33–36]. Regarding the decline rate of cortisol rhythm over the day, a flatter slope was demonstrated by three studies from the UK, US, and Canada, respectively [31, 32, 37]. By contrast, a Canadian study among paramedics did not find any relationships between shift work and cortisol secretion [38]. Nevertheless, we need to bear it in mind that the studies mentioned above were all with cross-sectional design. Due to the simultaneous assessment of shift work and cortisol it remains impossible to draw any causal inference based on such studies. In the past decade, only few longitudinal studies on shift work and cortisol trends over time have been published. Four studies examined recovery of cortisol diurnal pattern after shift work. Two Norwegian studies among offshore oil rig workers found that cortisol diurnal profiles were not recovered on day 7 and day 11 after 2-week 12-h night-shifts [39, 40]; while cortisol diurnal profile was recovered on day 5 after 5-day 8-h night-shifts among Chinese nurses [41], and it was recovered on day 7 after 7-day 8-h night-shifts among Danish police officers [42]. When the follow-up period is prolonged, the findings seem to become mixed. Kudielka et al. followed up a sample of German workers in an electronic manufacturing plant for 2 months, and they reported that AUC of salivary cortisol was significantly increased in the group of night shift [43]; similarly, a Dutch study in police officers suggested waking salivary cortisol began to rise from baseline to significantly higher levels at one-year follow-up after they started shift duty, then declined slightly at two-year follow-up [44]. However, Copertaro et al. did not confirm any significant association of shift work with serum cortisol among Italian nurses with one-year follow-up [45]. Overall, most of studies on shift work and cortisol had a major limitation in terms of their small sample sizes (usually < 50 subjects).

We therefore carried out a longitudinal study with one-year follow-up, in order to investigate the impact of shift work on the dysregulation of the HPA axis in terms of diurnal cortisol rhythm, in a sample of hospital physicians during residency who were at high risk of circadian disruption due to shift work schedule arrangement.

## Methods

### Study sample

At baseline, 1000 junior physicians in their 2nd or 3rd year of specialty training (residency) working in the wider area of Munich, Germany, were randomly selected to participate in a questionnaire survey, based on registration data of the Bavarian Chamber of Medical Doctors. A total of 621 completed questionnaires were returned (response rate 62.1%). Among the 1000 invited physicians, one third random sample, i.e., 334 subjects were also invited to participate in a series of saliva tests. Saliva samples were collected on 2 consecutive working days (which were not during or on the days after night shift), 4 times a day, including time points at waking (0 h), + 4 h, + 8 h, and + 16 h. Questionnaires were usually answered 1–3 days before saliva sampling. Of the 334 participants, 146 returned saliva samples (response rate: 43.7%). However, 57 samples had to be excluded due to steroid treatment, sampling error, low volume of saliva, or missing data on any 4 times of samples, which left valid saliva samples 99 subjects at baseline. After one year we followed up those 146 physicians who had previously responded both questionnaire survey and saliva samples. Among them, 91 physicians (response rate 62.3%) returned two-day saliva samples (same procedure as baseline). From those, 21 were excluded from further analysis due to the above mentioned reasons. Thus, valid cortisol measurements at both baseline and follow-up were available from 70 participants.

The study was approved by the Committee on Ethics of Human Research of the Medical Faculty, Ludwig-Maximilians University Munich (No. 016/04), and participants signed a letter of informed consent.

### Measures

In the baseline questionnaire, the physicians were asked “Do you take shift work (which is regular work outside normal daily working hours)?” with two response categories of “No” and “Yes”.

Saliva samples were collected using a small cotton swab with no additives (Salivette®, Sarstedt, Numbrecht, Germany). Participants were instructed to chew on the swab for 3 min, put the swab into the Salivette, note the time of sampling, keep the samples at ambient temperature and return them to the lab within 1 week. Cortisol remains stable for this period of time. Participants were asked to collect samples on 2 days. All saliva samples were stored in the laboratory at − 20 °C until cortisol analysis. Cortisol concentrations were determined employing a highly sensitive chemiluminescence immunoassay (Cortisol Saliva LIA, IBL, Hamburg, Germany). Endpoint detection was done using a chemiluminescence reader (Victor, Perkin Elmer, Rodgau, Germany). The assay shows a relevant cross reaction with the following steroids: Prednisolone (57%), 11-deoxycortisol (12%), corticosterone (2.5%), cortisone (2%) and prednisolone (1%). The lower detection limit of this assay is less than 0.16 ng/ml. To reduce error variance caused by interassay imprecision, all samples from one subject were assayed in the same run. In our hands, within-assay coefficient of variation was 7.2 and 5.4% at 0.8 and 5.0 ng/ml, respectively. Between-assay coefficient of variation at the same concentrations was 9.45 and 6.6%, respectively. Since cortisol data from two consecutive working days were available, we calculated the mean values to represent cortisol levels for the four sampling time points, waking (0 h), + 4 h, + 8 h, and + 16 h, respectively. Diurnal slope was produced by regressing cortisol values on sampling time, with anchorage on the waking point, to generate a mean rate of reduction in cortisol per hour [26, 31]. Total cortisol concentration over the day (AUC, ng/ml × hours) was calculated using a formula for area under the curve with respect to ground, based on all the four sampling time points [46].

In addition, information on age, gender, professional tenure, working hours, partnership, children, smoking, risky alcohol use, physical activity, overweight and obesity was also collected at baseline.

### Data analysis

Firstly, descriptive statistics were performed. Means and standard deviations (SD) were calculated for continuous variables, and relative frequencies for categorical variables. Due to the fact we draw on a subsample to investigate cortisol research, we also compared the baseline characteristics between cortisol-involved participants in the current analyses (*N* = 70) and cortisol-involved non-participants (*N* = 551) within the whole study population, using Student’s t-test for continuous variables or Chi-square test for categorical variables. Secondly, we further tested the differences of baseline characteristics and cortisol secretion levels between the groups without or with shift work. Thirdly, differences of cortisol levels on four sampling time points (waking (0 h), + 4 h, + 8 h, and + 16 h) at follow-up were examined by analysis of covariance adjusting for age, gender, professional tenure, working hours, partnership, children, smoking, risky alcohol use, physical activity, overweight and obesity at baseline; more importantly, we also controlled for cortisol levels at baseline to account for potential ceiling effect (i.e., upward change less likely for higher baseline scores) and floor effect (i.e., downward change less likely for lower baseline scores). Fourthly, multivariate linear regression was applied to examine longitudinal associations between shift work levels at baseline (independent variable) and diurnal cortisol pattern (slope and AUC) at follow-up (dependent variables). The results are shown as regression coefficients (β) with 95% confidence intervals (CI). These analyses were adjusted for biological factors (age and gender), work factors (professional tenure and working hours), family factors (partnership and children), and behavioral factors (smoking, risky alcohol use, physical activity, overweight and obesity) at baseline as well as baseline cortisol values, in order to assess robustness of associations. Finally, considering the nature of repeated measures in longitudinal studies, particularly when correlations at different time-points within-subjects need to be addressed, we also used mixed regression modeling to examine the longitudinal associations between shift work at baseline and repeated measures of cortisol levels over the one-year period of follow-up [47]. All analyses were conducted with SAS 9.4 SAS Institute Inc., North Carolina, US).

## Results

Table 1 shows the characteristics of the study samples (with valid cortisol data) at baseline (*N* = 70). The mean age equaled nearly 30 years, and 57% were women. Seventy-six percent were living with partners, and 83% had no children. Regarding health-related behaviors, the majority did not smoke, did not have risky alcohol use, were engaged in regular physical activity, and the vast majority had normal body weight. Half of the participants were in their first 2 years of medical residency, and mean working time was nearly 51 h/week. The cortisol-involved subjects (*N* = 70) were fairly comparable to the others who did not participate in corisol collection or did not have valid cortisol data (*N* = 551) with respect to socio-demographic, work-related, or behavioral characteristics.

**Table 1:** Characteristics of cortisol-involved participants and non-participants at baseline

Variables		Cortisol-involved participants	Cortisol-involved non-participants	*p*
		*N* = 70	*N* = 551	
Age (years)	(mean ± SD)	30.61 ± 2.63	30.51 ± 2.72	0.7777
Working hours per week	(mean ± SD)	50.86 ± 9.46	51.18 ± 9.66	0.7903
Gender	Men	30, 42.86%	273, 49.55%	0.2916
	Women	40, 57.14%	278, 50.45%	
Partnership	No	17, 24.29%	132, 23.96%	0.9515
	Yes	53, 75.71%	419, 76.04%	
Children	No	58, 82.86%	462, 83.85%	0.8325
	Yes	12, 17.14%	89, 16.15%	
Professional tenure	≤ 2 years	34, 48.57%	239, 43.38%	0.4094
	> 2 years	36, 51.43%	312, 56.62%	
Shift work	No	51, 72.86%	375, 68.06%	0.4151
	Yes	19, 27.14%	176, 31.94%	
Smoking	No	59, 84.29%	448, 81.31%	0.5442
	Yes	11, 15.71%	103, 18.69%	
Risky alcohol use	No	63, 90.00%	486, 88.20%	0.6583
	Yes	7, 10.00%	65, 11.80%	
Physical activity	Inactive	18, 25.71%	157, 28.49%	0.6263
	Active	52, 74.29%	394, 71.51%	
Overweight and obesity	No	60, 85.71%	446, 80.94%	0.3331
	Yes	10, 14.29%	105, 19.06%	

Difference determined by Student’s t-test or Chi-square test

Out of 70 study subjects, 19 (27%) physicians were engaged in shift work at baseline. Typical diurnal cortisol rhythm was observed, i.e., the cortisol level was highest at waking, and then declined gradually over the day. The overall cortisol slope was − 0.39, and AUC was 42.38. However, none of the study characteristics including all cortisol indicators was significantly different between physicians without and with shift work (Table 2).

**Table 2:** Characteristics of study subjects at baseline

Variables		Shift work: No	Shift work: Yes	*p*	Total (*N* = 70)
		*N* = 51	*N* = 19		
Age (years)	(mean ± SD)	30.57 ± 2.48	30.74 ± 3.05	0.8138	30.61 ± 2.63
Working hours per week	(mean ± SD)	50.82 ± 10.27	50.95 ± 7.08	0.9616	50.86 ± 6.46
Gender	Men	25, 49.02%	5, 26.32%	0.0878	30, 42.86%
	Women	26, 50.98%	14, 73.68%		40, 57.14%
Partnership	No	12, 23.53%	5, 26.32%	0.8090	17, 24.29%
	Yes	39, 76.47%	14, 73.68%		53, 75.71%
Children	No	43, 84.31%	15, 78.95%	0.5963	58, 82.86%
	Yes	8, 15.69%	4, 21.05%		12, 17.14%
Professional tenure	≤ 2 years	27, 52.94%	7, 36.84%	0.2307	34, 48.57%
	> 2 years	24, 47.06%	12, 63.16%		36, 51.43%
Smoking	No	42, 82.35%	17, 89.47%	0.4666	59, 84.29%
	Yes	9, 17.65%	2, 10.53%		11, 15.71%
Risky alcohol use	No	45, 88.24%	18, 94.74%	0.4201	63, 90.00%
	Yes	6, 11.76%	1, 5.26%		7, 10.00%
Physical activity	Inactive	13, 25.49%	5, 26.32%	0.9440	18, 25.71%
	Active	38, 74.51%	14, 73.68%		52, 74.29%
Overweight and obesity	No	45, 88.24%	15, 78.95%	0.3234	60, 85.71%
	Yes	6, 11.76%	4, 21.05%		10, 14.29%
Cortisol at waking, 0 h (ng/ml)	(mean ± SD)	7.90 ± 4.82	8.36 ± 3.55	0.7003	8.02 ± 4.50
Cortisol at +4 h	(mean ± SD)	2.90 ± 2.34	2.84 ± 1.46	0.9029	2.88 ± 2.13
Cortisol at + 8 h	(mean ± SD)	1.98 ± 1.19	1.59 ± 0.98	0.2078	1.87 ± 1.15
Cortisol at + 16 h	(mean ± SD)	0.91 ± 0.70	0.83 ± 0.65	0.6567	0.89 ± 0.68
Cortisol slope	(mean ± SD)	−0.38 ± 0.24	− 0.41 ± 0.17	0.6137	− 0.39 ± 0.22
Total cortisol AUC (ng/ml × hours)	(mean ± SD)	42.91 ± 20.55	40.95 ± 14.30	0.7037	42.38 ± 18.98

Difference determined by Student’s t-test or Chi-square test

**Fig. 1:**
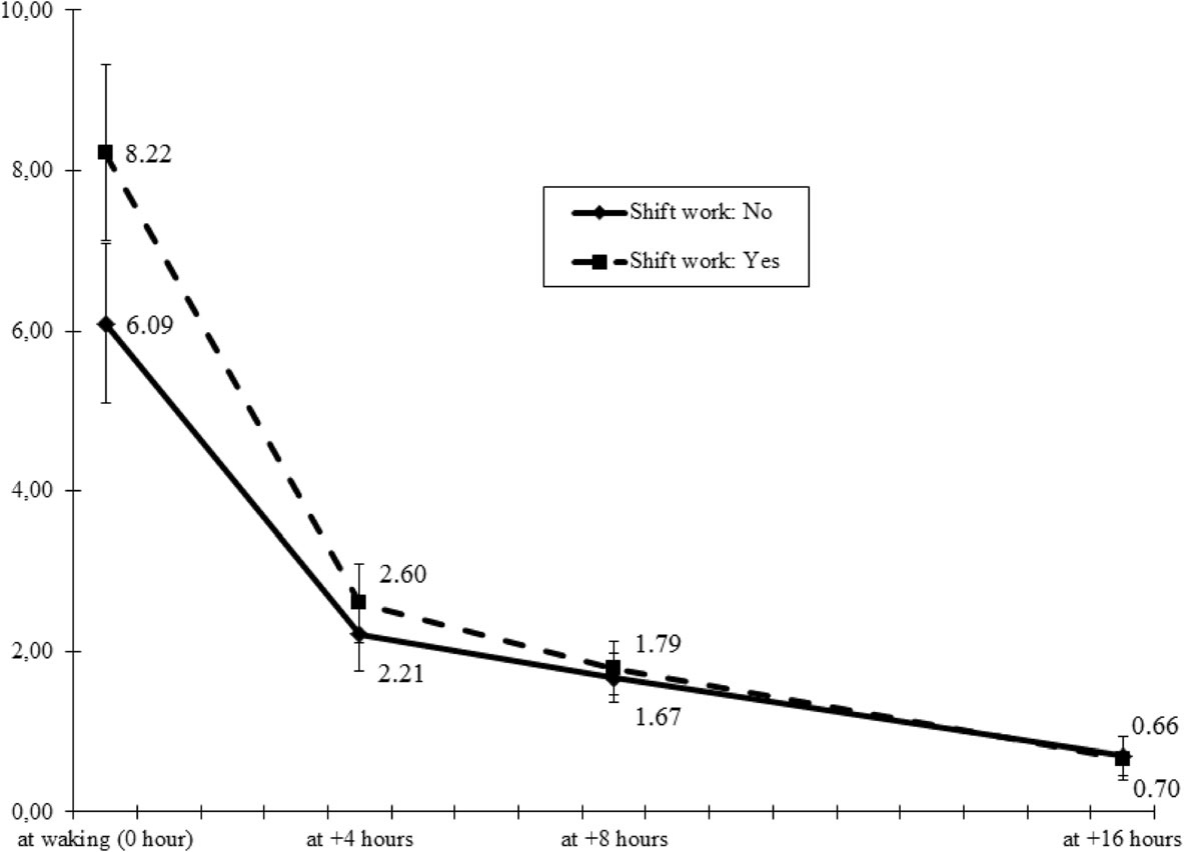
Diurnal pattern of cortisol secretion at follow-up according to shift work at baseline. (Solid line represents cortisol pattern at follow-up for physicians with shift work status “no” at baseline (*N* = 51); dashed line represents cortisol pattern at follow-up for physicians with shift work status “yes” at baseline (*N* = 19); Error bars represent standard errors of adjusted means (ng/ml) of four time points cortisol levels at follow-up)

Figure 1 illustrates diurnal cortisol rhythm at follow-up for the shift work group vs. the non-shift work group. After adjustment for socio-demographic, behavioral, work and family factors, as well as cortisol levels at baseline, waking cortisol was found to be significantly higher among physicians engaged in shift work (*p* < 0.01), whereas cortisol levels at the other three time points (+ 4 h, + 8 h, and + 16 h) did not differ by shift work status (details not shown).

The results of linear regression are shown in the Table 3. Throughout the adjustment procedure from models I to V, the associations remained stable. In the fully (final) adjusted model, shift work at baseline was associated with increased cortisol slope negatively by 0.12 (*p* < 0.01) and elevated total cortisol AUC positively by 6.64 (*p* < 0.05) 1 year later, indicating steeper slope and larger AUC over the day. Notably, mixed regression modeling, while taking the correlations of cortisol at baseline and at follow-up into account, exerted very similar findings (Table 4).

**Table 3:** Longitudinal associations between shift work at baseline and diurnal cortisol pattern at follow-up (*N* = 70)

Cortisol slope	Model I	Model II	Model III	Model IV	Model V
Shift work: No	0	0	0	0	0
Shift work: Yes	−0.09 (− 0.18, − 0.01)*	−0.11 (− 0.20, − 0.02) *	−0.11 (− 0.20, − 0.02)*	−0.12 (− 0.21, − 0.04)**	−0.12 (− 0.21, − 0.03)**
Total cortisol AUC	Model I	Model II	Model III	Model IV	Model V
Shift work: No	0	0	0	0	0
Shift work: Yes	5.36 (0.20, 10.52) *	6.19 (1.13, 11.25) *	6.33 (1.25, 11.41) *	6.71 (1.55, 11.86) *	6.64 (1.48, 11.79) *

Linear regression, β (95% CI), *p < 0.05, **p < 0.01

Model I: adjustment for biological factors (age and gender)

Model II: Model I + additional adjustment for work factors (professional tenure and working hours) at baseline

Model III: Model II + additional adjustment for family factors (partnership and children) at baseline

Model IV: Model III + additional adjustment for behavioral factors (smoking, risky alcohol use, physical activity, overweight and obesity) at baseline

Model V: Model IV + additional adjustment for cortisol secretion at baseline

**Table 4:** Longitudinal associations between shift work at baseline and diurnal cortisol pattern over one-year period of follow-up (*N* = 70)

	Cortisol at waking	Cortisol at +4 h	Cortisol at + 8 h	Cortisol at + 16 h
Shift work: No	0	0	0	0
Shift work: Yes	2.02 (0.68, 3.36)**	0.30 (−0.28, 0.88)	− 0.01 (− 0.38, 0.36)	0.02 (− 0.26, 0.29)
	Cortisol slope		Total cortisol AUC	
Shift work: No	0		0	
Shift work: Yes	−0.10 (− 0.17, − 0.03)**		5.84 (1.36, 10.32)*	

Mixed regression, β (95% CI), *p < 0.05, **p < 0.01

Adjustment for biological factors (age and gender), work factors (professional tenure and working hours), family factors (partnership and children), behavioral factors (smoking, risky alcohol use, physical activity, overweight and obesity) at baseline

## Discussion

The aim of our study was to examine the longitudinal impact of shift work on diurnal cortisol rhythm. Drawing on a sample of junior physicians from Germany, we found that shift work at baseline significantly changed the diurnal cortisol pattern at follow-up, in terms of higher waking cortisol, steeper slope and larger AUC, thereby predicting increased cortisol secretion at follow-up.

To date, cross-sectional studies generated contrasting evidence on the relationships between shift work and cortisol. As we mentioned above, in order to enable causal inference, longitudinal design is preferable. Therefore, the three longitudinal studies in the past decade deserve a close look. The Dutch study found an increase in waking salivary cortisol when police officers commenced duty of shift work 1 year later, and the effect was maintained for almost 2 years [44], while the German study, among industrial workers, suggested more cortisol secretion in saliva as larger AUC by changing work schedule from day shift to night shift for 2 months [43]. However, these two studies did not set an external reference group, that is, pre-and-post comparisons were actually conducted within subjects. The research design of the third study, conducted among Italian nurses, was quite similar to ours, with an external reference group (daytime work), with a one-year follow-up, and with adjustment for baseline cortisol values to take ceiling and floor effect into account [45]. Unfortunately, that study did not suggest any significant associations between shift work at baseline and cortisol levels at follow-up. Potential explanations might pertain to the approach to cortisol measurement, i.e., serum cortisol, because blood sampling itself represented acute stress reaction; and sampling point was one time only, i.e., 8:30–9:30 in the morning, resulting that the diurnal cortisol rhythm was impossible to be investigated [45].

Strengths of our study include its longitudinal study design to explore the potential causal association of baseline shift work and future changes in diurnal cortisol pattern which requires multiple sampling time points over the day. Furthermore, we recruited relatively larger sample size empowering our ability to detect fairly modest associations with statistical significance compared to most of earlier studies. To our knowledge, our study also produced first evidence of a longitudinal link between shift work and the cortisol slope, in addition to existing longitudinal findings on waking cortisol and total cortisol AUC [43, 44]. Nevertheless, our study also suffered several limitations. Firstly, just like in many previous studies on shift work, our assessment of shift work was not comprehensive enough [48]. In our study, participants only reported whether they were engaged into shift work. Additional information of interest had related to, (i) shift system on rotating or permanent schedule, regular or irregular arrangement; (ii) cumulative length of exposure; and (iii) shift intensity with time off (recovery days) between shifts [48]. Secondly, we need to mention the age difference and its effect on circadian impact of shift work. Both our study and one prior longitudinal study [44] identified higher waking cortisol by shift work, but many studies found shift work was associated with lower levels of waking cortisol [13, 33–36]. Also, we observed shift work steepened the decline rate of cortisol rhythm, whereas flatter slope was reported by other studies [31, 32, 37]. Age might serve as one potential explanation. The mean age was 30 years in our study and 27 years in the study by Lammers-van der Holst et al., respectively [44]. However, the mean age of most other studies was around 40 years or even older. Plenty of research has testified that, compared to younger people, waking cortisol is relatively lower and evening cortisol is relative higher in older people [49, 50]. This may imply that the pattern of shift work-caused diurnal cortisol change may differ in younger workers from ageing workers. The HPA axis is activated quickly in the morning and recovers in the evening (i.e., steeper cortisol slope) among young workers exposed to shift work; whereas the effect of shift work is obvious on evening cortisol but not on waking cortisol (i.e., flatter slope) among older workers. Certainly, future research on age, shift work, and HPA axis regulation is warranted. Thirdly, in our study, a cortisol sample was collected only on a single occasion in the morning, i.e., at waking. In psychoneuroendocrinological research, data on two sampling time points in the morning is generally preferred. If appropriately timed, those two assessments reflect the so-called “cortisol awakening response”, which is conceptualized as “a sharp increase in cortisol levels across the first 30-45 min following morning awakening” [51]. However, sampling accuracy and participants’ adherence are the major challenges in practice. Lacking data of cortisol awakening response is one main limitation regarding the research of diurnal cortisol rhythm. For future research, the consensus guidelines by an expert panel from the International Society of Psychoneuroendocrinology would be of great help [51]. Finally, as the current findings are restricted to young working people and one single occupation only, the ability of generalization to other age categories and occupations is limited. More longitudinal studies with larger sample size covering wider age range and various occupations are urgently needed in future.

As stated in a recent report from the US National Toxicology Program’s workshop on shift work at night, artificial light at night, and circadian disruption, “Understanding potential mechanisms and characteristics of light or shift work that are related to circadian disruption or biomarkers of disease may help identify interventions to protect public health.” [52] The available research evidence on shift work and diurnal cortisol rhythm would provide meaningful information to future interventions regarding work schedule management. For instance, according to a handful studies with respect to recovery of cortisol diurnal pattern after shift work, it would be desirable to allow for sufficient time periods off between shifts, such as 2 consecutive night shift days + 2 consecutive recovery days, or 5 + 5, 7 + 7, 14 + 14 arrangements [39–42]. Moreover, a review published in 2014 identified 44 intervention studies on health improvement among shift workers. In general, results support the benefits of fast-forward rotating shift schedules, i.e., morning-evening-night [53]. Specifically, compared to fast-backward rotating shifts, fast-forward rotating shifts exerted lower cortisol levels during the morning and night shifts [54].

## Conclusions

In conclusion, our longitudinal study, among junior physicians from Germany, supports the notion that shift work at baseline detrimentally affects the diurnal cortisol pattern 1 year later, specifically, higher waking cortisol, steeper slope and increased total cortisol secretion.

## Abbreviations

AUC: Area under the curve
CI: Confidence interval
HPA: Hypothalamic-pituitary-adrenal
SD: Standard deviation
UK: United Kingdom
US: United States of America
